# Privacy paradox and privacy calculus: the dilemma and trade-offs of privacy protection among Chinese middle-aged and elderly under digital stress

**DOI:** 10.3389/fpsyg.2025.1646272

**Published:** 2025-12-11

**Authors:** Jingya Hao, Cristina M Pulido, Yi Song

**Affiliations:** 1 Department of International Journalism and Communication, Beijing Foreign Studies University, Beijing, China; 2 Department of Journalism and Communication Sciences, Universitat Autonoma de Barcelona, Barcelona, Spain

**Keywords:** privacy, privacy paradox, the elderly, privacy calculus, digital stress

## Abstract

**Introduction:**

Digitalization has reshaped everyday life while creating new privacy and security risks, which are particularly acute for middle-aged and older adults who entered the digital era relatively late. This study examines how Chinese middle-aged and elderly individuals perceive digital privacy risks and navigate the tension between privacy protection and everyday digital participation under digital stress.

**Methods:**

Six focus group discussions were conducted with 25 Chinese participants aged 45 and above, and the transcripts were analyzed using inductive qualitative content analysis.

**Results:**

Findings reveal that while older adults show a high level of privacy awareness, their protective behaviors are shaped by culture, gaps in digital literacy, and structural dependence on digital infrastructures. Under digital stress, these constraints give rise to a privacy paradox in which individuals remain vigilant yet make strategic compromises to sustain digital participation.

**Discussion:**

The findings suggest that the privacy paradox among Chinese middle-aged and elderly users is less an attitudinal inconsistency than a structural dilemma shaped by cultural norms, digital literacy gaps, and institutional dependence on digital infrastructures, highlighting the need for supportive designs and policies that reduce digital stress and strengthen privacy protection capabilities in later life.

## 1 Introduction

With the rapid development of technology and the widespread use of smartphones, digitalization is substantially reshaping people’s daily lives. According to the 54th Statistical Report on China’s Internet Development, the number of netizens in China has reached approximately 1.1 billion, covering 78% of the population (China Internet Network Information Center [CNNIC], 2024). Although younger generations constitute the majority, elderly participation in digitalization has significantly increased in recent years. The report indicates that individuals aged between 50 and 59 account for 15.2% of new netizens, while those aged 60 and above account for 20.8% (China Internet Network Information Center [CNNIC], 2024).

However, increased connectivity has also exposed older adults to new forms of digital vulnerability. In recent years, cases of online scams, fraudulent investment links, and personal data misuse have frequently targeted this demographic. According to the Practice and Effectiveness Report on Improving Digital Literacy among Middle-aged and Elderly People released by the Renmin University of China Population and Development Research Center (2024), digital fraud has become one of the most prominent risks faced by elderly internet users. Similarly, the 2023 Mobile Payment Security Survey Report published by China UnionPay indicates that more than 70% of older users have encountered or nearly encountered online scams (China UnionPay, 2023). The ongoing digitalization of everyday life has moved many essential activities, such as communication, financial transactions, and public services into online spaces. Those experiences contribute to a growing sense of digital stress, or a persistent tension between the need to be included and the fear of privacy breaches and financial loss. Financial privacy, in particular, has become a salient domain where these contradictions are visible, as online payment, healthcare, and social platforms all require personal data disclosure.

While previous studies have examined the “privacy paradox,” the inconsistency between high privacy concern and low protective behavior (Acquisti et al., 2015), most have focused on younger users or Western contexts. In China, however, rapid digitalization and collectivist cultural values create a distinct context for understanding privacy behavior among older adults. Moreover, the classical privacy calculus framework (Culnan and Armstrong, 1999), which views privacy decisions as rational cost and benefit trade-offs, has rarely been tested under the emotional and structural constraints faced by aging users in China.

Therefore, this study investigates the dilemma and trade-offs of privacy protection among Chinese middle-aged and elderly individuals under digital stress, integrating privacy paradox and privacy calculus perspectives. Specifically, it examines how cultural framing, digital literacy, and perceived stress shape privacy-related decisions, and what adaptive strategies older users develop to balance risk and participation in a rapidly digitizing society.

## 2 Literature review

### 2.1 Privacy paradox and privacy computing

Privacy concerns, also known as privacy attention or privacy worries. In social media activities, users’ privacy protection behaviors can generally be categorized into two types (Chen and Wen, 2020). The first is through preventive privacy protection behaviors (preclusion), that is, treating social media as a public stage and choosing non-controversial and non-aggressive public topics to ensure that the content posted is as consistent as possible with the positions of followers. The second privacy protection behavior mainly involves privacy settings operations, such as avoiding identifiable personal information, differentiating content visibility, and posting through different groups or accounts (Stutzman et al., 2013; Vitak, 2012). People often express concerns about privacy, but do not change their privacy-disclosure behavior for various reasons. This phenomenon is known as the “privacy paradox,” which refers to “the discrepancy between people’s expressed privacy concerns and their actual privacy exposure behavior” (Oetzel and Gonja, 2011). The term “privacy paradox” was first used by Brown (2001) in a general context to describe the inconsistency between people’s privacy attitudes and behaviors. Later, Barnes (2006) introduced the concept in the context of social media in her article “*A Privacy Paradox: Social Networking in the United States*” published in *First Monday* (Shen and Qiu, 2021). Since then, numerous related studies have been conducted within academia, mainly through quantitative research to identify the existence of the privacy paradox and explore influencing factors.

In the Chinese context, research on privacy paradox has primarily centered around WeChat. WeChat, developed by Tencent, is one of China’s most pervasive digital platforms, integrating instant messaging, social networking (via “Moments”), mobile payment, and public information services. Its multifunctional design combines interpersonal communication, financial transactions, and media consumption within a single ecosystem. These features make WeChat a particularly relevant context for examining online privacy behaviors and disclosure patterns among Chinese users. For example, Xu et al. (2018) investigated the privacy concerns, privacy cognition, privacy worries, and privacy protection of college students in six universities in China through a survey on their WeChat usage and examined the relevant conditions and their interrelationships. The research results proved that there was a significant “privacy paradox” phenomenon among college students in their use of WeChat, that is, on the one hand, they were worried about the leakage of their personal privacy, but on the other hand, they actively disclosed a large amount of privacy information. In addition, taking WeChat as an example and focusing on the Internet generation, Yuan and Hou (2016) employed questionnaire surveys and data mining techniques using a decision tree classification algorithm to identify the presence of the privacy paradox phenomenon among WeChat users. The study revealed that personal characteristics, privacy protection awareness, and WeChat usage habits significantly influence privacy paradox behaviors.

Beyond WeChat, the privacy paradox has also been observed on global platforms such as Facebook and Twitter. For instance, Taddicken (2014) found in research on online social network users in Germany that privacy concerns have little impact on self-disclosure behaviors in practice. Similarly, Kwok Choon (2018) examined privacy practices across Facebook and Twitter and found that privacy concerns are context-dependent. On Facebook, users perceived privacy violations as potentially damaging to their reputation and professional identity, whereas on Twitter, visibility and exposure often outweighed privacy considerations. These findings further confirm that the privacy paradox varies across platforms and social contexts, reflecting distinct norms of self-presentation and perceived risk.

Additionally, There have been numerous studies that have attempted to explain and explore the causes of the “privacy paradox.” For instance, Acquisti (2004) examined the causes of consumers’ privacy paradox behavior in the e-commerce environment from the perspective of bounded rationality, suggesting that consumers might selectively ignore potential risks due to a sense of luck, thereby engaging in behaviors inconsistent with their attitudes toward privacy. Zhu et al. (2017) employed privacy calculus theory and combined with the Theory of Planned Behavior (TPB), explored the causes of the privacy paradox phenomenon. The study found that users’ risk perception positively affects their privacy concern level, thereby reducing their intention to disclose personal privacy. However, when users perceive higher potential benefits, their willingness to disclose privacy significantly increases, and eventually leading them to take disclosure actions. This process well explains the underlying mechanism of the privacy paradox phenomenon. From the perspective of rational decision-making, the causes of the “privacy paradox” can be generally divided into two directions: the perspective-oriented theory and the behavior-oriented theory (Liu and Deng, 2018). The perspective-oriented theory holds that the main cause of the privacy paradox is due to the cognitive limitations of users. Supporters of the behavior-oriented theory, however, believe that users, unaffected by subjective factors, will rationally weigh the benefits they can obtain against the risks of privacy disclosure and make decisions that maximize benefits or minimize risks. That is, users will, through a certain “privacy calculus,” rationally weigh the risks and benefits of privacy disclosure, leading to a contradiction between their final behavior and their concerns (Liu and Deng, 2018). The theory of “privacy calculus” was first proposed by Culnan and Armstrong and initially applied in the e-commerce field. This theory holds that individuals will conduct a cost-benefit analysis when disclosing information, and only when the expected benefits are greater than or equal to the potential risks will they choose to disclose information (Culnan and Armstrong, 1999; Culnan and Bies, 2003). In the theory of privacy calculus, the decision-making process of information disclosure is described as a cognitive process based on full information, autonomous judgment, and careful consideration (Geng, 2024). Due to the complex and diverse elements of gains and losses in real life, in addition to the considerations of benefits and risks, there are other factors that affect privacy decisions, such as default settings and limited choices (Geng, 2024). That is, in many cases, users are forced to disclose their personal information either intentionally or involuntarily (Zhang et al., 2018).

However, more recent studies have questioned whether the so-called “privacy paradox” should be interpreted merely as a rational inconsistency. Hoffmann et al. (2016) proposed the concept of privacy cynicism, describing a coping mechanism whereby individuals, overwhelmed by privacy threats, develop a sense of resignation and rationalize their continued engagement with digital services despite serious concerns. Similarly, Solove (2021) challenges the very foundation of the “privacy paradox” by arguing that it is a myth created by faulty logic. He contends that privacy attitudes are general in nature, while behavioral studies capture decisions made in specific contexts. Therefore, the apparent inconsistency between privacy attitudes and behaviors reflects contextual decision-making rather than a genuine paradox.

### 2.2 The middle aged and older adults in the digitized society

In research on online privacy, age has been widely discussed as a significant factor. In the face of the rapid digitalization process, the middle-aged and elderly, as late adopters of digital technologies, encounter significant barriers and heightened stress in technology use compared to younger user groups (Berkowsky et al., 2018). Although digital technology is increasingly widely used among the elderly and has been shown to help alleviate their depression and anxiety, provide social support, enhance health literacy, and improve social integration (Jiang et al., 2024), they still face significant obstacles when using digital technology, especially when it comes to concerns about personal privacy leakage and property security, where anxiety is more pronounced (Wang, 2024). In the digital age, the middle-aged and elderly are often marginalized as the “digital marginalized group,” facing “digital age discrimination.” This discrimination is manifested in the design, development, deployment, and evaluation of digital technology, and the scarcity of elderly application and training data further exacerbates this systemic age bias (Hu, 2024).

In a study focusing on middle-aged and elderly people who do not use social media (Lüders and Brandtzæg, 2017), it was found that privacy and security concerns are one of the main reasons why they refuse to use social media. In another study that included middle-aged and elderly people (Quan-Haase and Elueze, 2018), it was discovered that both social media users and non-users share similar privacy concerns, with the most significant being unauthorized access to personal information and information misuse. The difference lies in that the elderly non-users protect themselves by avoiding social media, while the elderly users protect themselves by limiting the information they share. In a study by Shan and Yi (2021), which investigated the influencing factors of information privacy concerns of social media users, it also verified that age has a significant negative impact on social media information privacy concerns, meaning that privacy concepts change with age, and the older they are, the less they can withstand the potential privacy risks. Additionally, past experiences have been proven to have a significant impact on privacy awareness and privacy protection behaviors, especially negative experiences. Yang (2013) found that although users are very concerned about privacy issues such as data collection and unauthorized secondary use, it is the negative experiences of past privacy leaks that can truly prevent behavior.

Some qualitative studies have provided deeper insights into how older adults perceive and manage privacy in technology-mediated environments. For instance, Birnholtz and Jones-Rounds (2010) conducted interviews with 30 seniors, their caregivers, and relatives to explore how physical space, temporal routines, and social interaction shape perceptions of privacy and independence in everyday life. Similarly, Elueze and Quan-Haase (2018) examined older adults’ privacy attitudes in their digital lives and found substantial heterogeneity among individuals. While their levels of concern varied, common worries included spam, unauthorized access to personal data, and information misuse. The study also emphasized the importance of offering privacy literacy training tailored to the needs of this demographic group.

It can be observed that although there is a considerable amount of research on privacy issues in social media usage, studies from the perspective of middle-aged and elderly people in the Chinese context are still insufficient. Moreover, previous research mostly adopted quantitative methods, lacking in-depth analysis of the underlying mechanisms behind their privacy perceptions and behaviors. Therefore, this study adopts a qualitative approach to explore how Chinese middle-aged and elderly individuals perceive and respond to digital privacy risks in their everyday online activities. Specifically, it aims to answer the following research questions:

1. How do middle-aged and elderly people in China perceive and experience digital privacy risks in their everyday online interactions?
2. How do they respond to and manage digital privacy protection?
3. How does digital stress influence their privacy-related attitudes, behaviors, and decision-making processes?

## 3 Methodology

Focus group discussion is an important qualitative technique that is widely used in communication studies (Morgan, 1997). While quantitative research provides general statistical information, qualitative research exploration allows us to understand and explain the reasons (Tenny et al., 2022). To have a more insightful understanding of elderly adults’ experience, this study adopts a qualitative research design.

### 3.1 Recruitment strategy and ethics

Participants were recruited using a snowball sampling strategy in urban China. Initial participants were identified through personal contacts and community networks, and then recommended additional eligible participants. There were no specific inclusion criteria regarding digital media experience; participants only needed to be 45 years or older.

Prior to data collection, the study protocol was reviewed and approved by the Institutional Review Board of the University. All participants were fully informed of the research purpose, confidentiality procedures, and their rights to withdraw at any time before providing consent. Written and informed consent was obtained from all participants before participation. Before each focus group discussion, the moderator also provided a brief oral introduction to the purpose of the study, and the confidentiality and voluntary participation principles.

### 3.2 Participants

In total, 25 participants in 6 groups participated in focus group discussions. In addition to age, participants represented a range of educational backgrounds (from high school to university) and varying levels of digital literacy, including frequency of digital use, use of digital payments, and bank account linkage to online platforms.

Table 1 presents the demographic and digital behavior characteristics of the 25 participants. The sample covered a broad age range, with a majority aged between 50 and 59. Most participants reported using WeChat Pay, and had linked their bank accounts to online payment platforms. About half of the participants had children living nearby. The sample included more male than female participants, reflecting the characteristics of the recruitment network and the snowball sampling strategy. Given the exploratory nature of this study, this gender imbalance is not expected to substantially affect the interpretation of the findings.

**TABLE 1:** Demographic and digital behavior characteristics of participants (*N* = 25).

Variable	Category	N	%
Age	45–49	7	28
	50–59	14	56
	60–69	2	8
	70 and above	2	8
Gender	Male	17	68
	Female	8	32
WeChat Pay use	Yes	21	84
	No	4	16
Bank card linked	Yes	17	68
	No	8	32
Children nearby	Yes	13	52
	No	12	48

Because participants were recruited through snowball sampling, grouping basically followed natural social networks, which means participants who were familiar with each other were assigned to the same group. This approach facilitated more open and comfortable discussion, which is particularly valuable in the Chinese context where participants may be more reserved in conversations with strangers.

### 3.3 Procedure

All focus groups were conducted face-to-face in a community activity room and a meeting room located in two cities. The environment was quiet and familiar to participants, which helped create a comfortable atmosphere and encouraged open sharing.

A semi-structured interview format was adopted, and all sessions were moderated by the lead author, who has professional training in qualitative interviewing and experience working with older adults. The moderator shared similar cultural background and linguistic familiarity with the participants, which helped build rapport and facilitated natural conversation. Discussions covered topics such as the perception and understanding of privacy, strategies for protecting online privacy, and attitudes toward digital society. The full focus group interview guide is provided in Appendix A.

Each session lasted approximately 35–60 min depending on participant engagement. and all the conversations were audio-recorded. After the interviews, all recordings were transcribed verbatim, and colloquial expressions were standardized where necessary.

Data saturation was monitored throughout the coding process. Saturation was reached after the fifth focus groups, when no new codes or themes emerged from the data, and one additional session was conducted to confirm saturation.

### 3.4 Data analysis

The transcripts were analyzed using NVivo 12, and participants’ identities were anonymized to ensure confidentiality. A line-by-line coding approach was applied to inductively identify themes emerging from the data. Although no predefined coding framework was applied, the analysis was guided by the principles of inductive qualitative content analysis, allowing categories and themes to emerge from the data rather than being imposed *a priori* (Mayring, 2000).

The coding was conducted by one coder using an iterative approach. To enhance trustworthiness and minimize potential bias, the author conducted multiple iterative reviews and engaged in peer debriefing with two colleagues experienced in qualitative research.

## 4 Results

Through inductive coding, three overarching themes were identified:

(1)    Perceived Privacy Risks, which capture participants’ sense of exposure, concerns about self-expression, and heightened anxiety around financial security.
(2)    Protective behaviors, referring to the coping strategies participants used to manage perceived risks.
(3)    Digital Stress, which reflects the paradoxical situation in which older adults continue to use digital platforms despite their concerns, adopting various strategies to balance risk and convenience.

A summary of the coding structure is shown in Table 2.

**TABLE 2:** Main theme and subtheme.

Main theme	Subtheme	Description	Illustrative quote
Perceived privacy risks	Exposure and helplessness in the big data era	Sense of surveillance and lack of control	“In the era of big data, we are like running naked.” (G1-3)
	Prudent self-expression	Perceived risk of being misunderstood or criticized, and of attracting unwanted attention or unnecessary trouble	“I didn’t post a single photo this time, mainly considering what others might think.” (G1-2)
	Financial privacy and risk anxiety	Fear and distrust of digital payments, shaped by media and family warnings	“My son warned me not to link my card… if the phone gets stolen, my money is gone.” (G4-4)
Protective behaviors	Privacy calculus and strategic compromise	Active risk management through partial use, limiting funds, and relying on family	“I keep only a small amount of money in my account.” (G1-4)
Navigating digital stress	The privacy paradox	High concern but continued use of digital tools due to necessity	“It’s not a matter of choice but of survival.” (G1-2)

### 4.1 Perceived privacy risks

#### 4.1.1 Exposure in the era of big data

Across all groups, participants expressed a strong sense of exposure and powerlessness in the face of big data. Most participants felt that their personal information was constantly being exposed and had little confidence in their ability to protect it.

“*In the era of big data, we are like running naked. Everything is known. Where you go, what you spend, all is clear.*” (G1-3, male, 51)

This is how Mr. Zhang evaluated online privacy, and this view quickly resonated with other members of the interview group. In the era of big data, various kinds of personal behaviors are recorded, greatly increasing the risk of leakage of personal information and privacy. Mr. Shi from another group also described the uncontrollability of information in the era of big data in this way:

“*Nowadays, I increasingly feel that there seems to be no privacy at all. Your personal information, your phone numbers, are difficult to control. Sometimes you may receive a WeChat friend request, with your name called out, saying that someone asked him to contact you (but it turned out to be from a scammer).*” (G5-2, male, 70)

In the interviews, except for a few respondents who expressed indifference toward “protection,” claiming they had “no significant privacy,” most respondents held a negative attitude, believing that privacy protection was “unachievable and passive.” This sense of uncontrollable exposure demonstrates that older adults do not perceive privacy loss as an isolated personal issue, but as a structural consequence of digital systems, which reinforces feelings of helplessness and fatalism toward technology.

#### 4.1.2 Prudent self-expression

In group discussions, many participants reported high concerns in self-expression, indicating that they are very cautious in self-expression on WeChat Moments (a feature similar to a social media feed where users can post updates and share with friends) and often self-censor the content they post to avoid speculation or unwanted attention, fearing that it might lead to others’ speculation or unnecessary trouble. As one of the participants (G1-2, male, 56) expressed that he used to enjoy sharing travel photos on WeChat but stopped due to concerns over potential negative feedback:

“*I like taking photos of beautiful scenery or places I’ve visited and sharing them with everyone. Earlier this year, I went on a trip but didn’t post anything (on WeChat Moments).*” (G1-2, male, 56)

As another participant laughed and added, and the participant (G1-2, male, 56) further explained:

“*Otherwise, others would know you went traveling again.*” (G1-1, male, 55)

“*Our trips are self-funded, and there was nothing wrong with it. However, some people advised me not to post travel photos as it might give a bad impression. So, I didn’t post a single photo this time, mainly considering what others might think, although I still want to share with others.*” (G1-2, male, 56)

Regarding the more frequent and straightforward expression style of younger people on WeChat Moments, one participant, 70-year-old Mr. Shi, held a rather negative view, believing that young people’s behavior is “immature and irrational.” He expressed:

*“Posting comments and moods casually on WeChat Moments is like using WeChat as a place to vent emotions. Sharing every move not only fails to solve real problems but may also raise safety concerns.”* (G5-2, male, 70)

These ideas were supported by most of the participants, reporting that they prefer selective or minimal self-expression instead of sharing posts freely.

This phenomenon reflects the older adults heightened privacy awareness. Compared to younger people, their self-expression is more constrained and defensive. This also indicates their cultural expectations around modesty and social harmony. Rather than using privacy settings to protect visibility, older adults tend to pre-empt judgment by self-censoring, showing that privacy protection is practiced through social caution rather than technical control.

#### 4.1.3 Financial privacy and risk anxiety

Beyond concerns about social self-expression, privacy issues were particularly salient in financial security. During the interviews, respondents exhibited a high sensitivity to financial risks. This apprehension primarily stems from two main sources: first, fear induced by media reports on fraud cases and frequent warnings from their children; second, anxiety arising from a lack of experience, leading to fear of the unknown or unfamiliar situations.

For the middle-aged and elderly, although new media such as mobile phones and WeChat have become integrated into their daily lives, television still holds significant influence. Many people still have the habit of watching the 7 PM news broadcast every day, and the interviewees expressed a high level of trust in the information presented on TV. During the interviews, a considerable amount of fear regarding financial risks stemmed from television reports on fraud cases. Numerous programs detailed incidents where victims’ money was transferred through sophisticated technical means. Participants were able to recount these cases in detail, creating a lively discussion atmosphere. For example, one participant described:

“*When you buy something at the supermarket and pay, a scammer behind you opens their phone, scans something, and your money disappears immediately. That was exposed by China Central Television (CCTV). CCTV also revealed another case where hotel staff collaborated with criminal groups, taking overdraft card and swiping it; immediately, the card number was captured below. Then, when you enter your password, a camera above records it, and your bank card gets cloned*.”

Furthermore, Family warnings further amplified this sense of threat. Much of the anxiety regarding financial risks stems from the overprotective warnings of their children. Many participants reported being “frightened” by their children in the past, which caused them to fear engaging in financial operations. One participant said,

“*Before I started using a smartphone, my son “scared” me: “You’d better not do this, better not do that.” My child warned me that if I link my bank card to WeChat and my phone gets stolen, my money could be transferred without permission*” (G4-4, female, 55).

This overprotection paradoxically intensified their fear of technology. Many described digital financial tools as a “black box” they could not fully understand but felt forced to use. This situation fuels their privacy concerns and heightens their fear of technology. Such fear not only hinders their integration into the digital society but also exacerbates the digital divide (McDonough, 2016). Their lack of digital experience makes it difficult to alleviate technological fears through hands-on practice, leading instead to deeper worries. As one participant expressed,

“*I’m afraid to try, afraid to make mistakes, sometimes it makes me scared to do anything at all*” (G4-1, female, 51).

Thus, we can see that financial privacy concerns are culturally, emotionally, and structurally reinforced: media scandals amplify threat perception, family warnings intensify fear of making mistakes, and unfamiliarity with digital interfaces prevents experiential learning.

### 4.2 Protective behaviors

1. Despite strong privacy concerns, participants did not reject digital platforms altogether. Instead, they adopted a range of pragmatic strategies to reduce perceived risks while maintaining access to digital services. These findings address RQ2 by demonstrating that older adults do not passively accept privacy risks. Instead, they employ incremental, pragmatic, and socially supported strategies to reduce exposure.

(1) Reducing online visibility

1. Most participants avoided complex privacy settings and used simpler behavioral strategies, such as posting less frequently, carefully selecting content, or stopping posting entirely.

For many young people, complex privacy settings already pose a challenge, and for middle-aged and elderly individuals, these settings may be even more complicated. In the interviews, some participants set the visibility time for their Moments, but very few made any distinction in the visible audience of their Moments. It is evident that, unlike young people who typically use settings such as grouping to control who can access their information, middle-aged and elderly people mainly avoid potential risks through more direct and simple methods, such as reducing the frequency of posting, carefully selecting content, or choosing not to post at all.

(2) Partial use of financial functions.

Although many participants expressed concerns about financial risks associated with digital payments, especially heightened vigilance regarding privacy leakage when linking bank cards, nearly all of the participants use WeChat Pay. As one participant remarked,

“*I do worry about the security of my bank card and wouldn’t link it to my WeChat account. But in the end, you still have to use these tools*” (G1-1, male, 55).

Throughout this process, middle-aged and elderly individuals demonstrate a high sensitivity to privacy risks alongside strategic compromise. They do not fully accept the technology, but, in the game with privacy risks, they tried to avoid risks through “partial use” and achieved a balance between risk avoidance and self-protection under digital stress. For instance, many respondents chose not to link bank cards. However, they use alternative methods in digital payments. The convenience of WeChat Pay and its wide application have made it an indispensable tool in daily life, such as the popular “red envelope” function. WeChat Pay is particularly important for the middle-aged and elderly group as it not only simplifies the payment process but also strengthens interaction with family and friends. However, due to its “non-physical nature,” elderly people have high concerns about privacy and financial risks.

“*I have concerns about bank cards, so I won’t link it to my WeChat account. If your bank information is leaked, you will lose all your money, and it will never be found.*” (G3-1, male, 54)

(3) Informal “digital currency support system.”

Some middle-aged and elderly people adopt more flexible approaches to using digital payment functions, a common coping strategy was exchanging cash with children or friends to recharge their WeChat balance, allowing participation without exposing bank accounts. By mobilizing the help of relatives and friends, they have built an informal “digital currency support system,” which not only meets their needs for digital payments but also reduces the risk of directly linking bank cards. This continuous adjustment of privacy prevention and practice is specifically manifested as the negotiation between privacy concerns and digital stress in the use of digital technology.

(4) Limiting financial exposure.

Moreover, many middle-aged and elderly people adopted a “limit strategy,” that is, limiting the amount of funds in the linked bank card to a low level to reduce potential financial risks. Almost all respondents who had linked bank cards said that they would strictly control the balance on the card. For example, some respondents kept only a small amount of money in the linked bank card, while others tried to avoid linking credit cards or salary cards.

“*I transfer most of my salary out every month and only leave a small amount just in case.*” (G1-4, male, 55)

These strategies illustrate a form of privacy calculus, also a negotiated balance between convenience and perceived vulnerability, which is enabled by practical adjustments rather than technical mastery.

### 4.3 Digital stress

Even though these coping strategies help reduce perceived risks, participants did not feel fully protected. In terms of financial risk protection, although they have a high level of concern about financial risks such as information leakage and fraud related to QR code payments, many still use WeChat Pay in practice. One participant said,

“*I definitely have concerns about my bank card, so I don’t link them. Once something happens, your money just drains away. Once it is stolen, your money just drains away and you will in deep trouble, even the police can’t help you.*” (G3-1, male, 54)

However, almost all interviewees had adapted to using WeChat Pay out of necessity. As one participant reflected,

“*Worrying too much is pointless. If you really encounter trouble, it’s just bad luck*” (G1-1, male, 55).

Another remarked,

“*Everything carries risks. To be honest, whether it’s bankbooks or bank cards, or cash or other things, which one doesn’t have risks? So, people have gradually got used to it.”* (G1-1, male, 55).

Regarding how to better solve this problem, in addition to improving their own usage and identification abilities, many people mentioned the national regulatory level, believing that the state will carry out effective supervision and that the designers of various apps will continuously improve them.

“*I feel that from the perspective of the designers of Alipay or WeChat Pay, they have been gradually improving it, and the government is also supervising it.”* (G1-2, male, 56)

Since there is a high level of concern about privacy and an apparent inability to effectively protect it, is refusal to use digital technologies a viable way to avoid this dilemma? The answer is probably no. In recent years, digitalization has been like a huge machine, forcing everyone to move forward and join the digital process. Technology and digitalization have brought people great convenience, but when embracing technology, it is also necessary to pay attention to those who are forced into the digital process, among whom there are many middle-aged and elderly people, especially the elderly. In the interviews, although the respondents recognized the convenience brought by WeChat and WeChat Pay, many of them started to use them passively under the stress of digitalization, especially WeChat Pay. As one respondent stated:

“*New things must be accepted; eventually, you have to accept them. You might refuse at first, but in the end, acceptance is inevitable. At first, there was WeChat but no WeChat Pay, but now it’s necessary to take this step*” (G1-2, male, 56).

For new technologies, individuals may not have much choice, because *“it’s not a matter of choosing whether to accept or not, but rather a matter of survival.”* (G4-5, female, 57).

For example, the experiences of the following participants illustrate this digital stress faced by the middle-aged and elderly population:

G1-1: *Last year, I went to Beijing for a trip. Everywhere, they didn’t accept cash; it was all QR code payments. If they don’t accept cash, what’s the point of carrying cash, right?*

G1-3: *Last year, I went to a restaurant. There were no waiters, no menus. There was only a QR code on the table, and when you scanned it, the menu popped up. After a while, the food was brought over. Only one person was serving the dishes, and nothing else. At first, we were all waiting for the menu, but nobody came to us.*

Such scenarios illustrate how digital infrastructures normalize cashless transactions, meaning that opting out of digital tools may result in practical exclusion rather than enhanced protection.

## 5 Discussion

In recent years, although privacy protection issues have received increasing attention, how to protect privacy in practice remains an urgent problem to be solved., especially for the elderly. This study reveals that while middle-aged and elderly users in China exhibit a high level of privacy awareness and concern, their actual protective actions are limited, shaped by cultural norms, digital literacy gaps, structural pressures, and emotional factors.

### 5.1 Cultural framing, media literacy, and social support

1. The findings highlight how cultural norms and media literacy gaps shape the privacy behaviors of middle-aged and elderly users in China. This aligns with Liang et al’s. (2017) global privacy study involving 3.3 million Twitter users, which found that users in collectivist societies disclose less personal information and adopt fewer technical privacy protections compared to those in individualist societies. This stands in sharp contrast to the more open and direct modes of expression typical of Western individualistic cultures and expressive patterns typical of Western individualistic cultures, underscoring that privacy behaviors are not purely technical choices but are culturally embedded practices.

In Chinese cultural traditions, values such as modesty, prudence, and self-restraint are deeply embedded. Confucius emphasized being “slow in speech but prompt in action.” In the family precepts of Zhu Bolu from the Ming Dynasty, it is also stated that “do not speak much in dealing with the world; too much talk will lead to mistakes.” These values shape how individuals perceive and navigate digital spaces. For older generations in particular, influenced by historical and educational contexts, self-censorship has evolved from a habit into a default mode of digital interaction.

This cultural backdrop explains why many participants were reluctant to post on WeChat Moments despite having the technical means and media literacy to do so. Their concerns over potential gossip, misinterpretation, or unintended visibility reflect an intersection between privacy concerns and cultural restraint. This finding extends existing privacy literature by highlighting that cultural factors shape not only what people disclose but also how they choose not to disclose.

However, cultural framing alone cannot fully explain their limited privacy protection practices. Media literacy also plays a crucial role. Participants in our study reported that accumulating digital experience helped them gradually overcome the fear of technology. Faced with the “black box” of digital payment, many middle-aged and elderly individuals believe that rather than succumbing to fear, it is better to “open the box” and try. Through a certain degree of trial and error, they gradually adapt to the technology. Accumulated digital experience thus serves as a way to reduce perceived risk and anxiety.

Moreover, intergenerational support emerged as an informal but crucial mechanism for overcoming literacy barriers. In the digital era, the absolute authority of the older generation is gradually diminishing, while the younger generation, as digital natives, is becoming the new digital authority (Xie and Zhang, 2024). As Berkowsky et al. (2018) pointed out, children and close relatives play a critical role in helping middle-aged and elderly adults understand and use new technologies. Their support not only helps alleviate anxiety about new technology but also enhances their trust in digital tools. This relational dimension of digital literacy reveals that privacy behaviors are not purely individual decisions but are embedded in family and social contexts.

Taken together, these findings suggest that privacy behaviors among older Chinese adults are shaped by a cultural background, constrained by digital literacy, and mediated by social support. This intersection sets the stage for understanding their ambivalent and often constrained privacy choices in the face of digital stress, laying a foundation for the subsequent analysis of the privacy paradox and strategic compromise. In this sense, digital stress among older adults can also be understood as a manifestation of the broader digital divide, not only in access, but in skills, confidence, and meaningful participation.

### 5.2 Privacy paradox under digital stress

1. Although middle-aged and elderly users exhibit strong concerns about privacy, their actual digital practices often contradict these attitudes. This contradiction exemplifies the privacy paradox, where individuals express high levels of privacy concern but simultaneously disclose personal information online (Acquisti et al., 2015). Among older users, this paradox is particularly pronounced due to the combined effects of limited privacy protection capabilities and digital stress—a persistent pressure to adapt to rapidly digitizing social environments.

In social media activities, users typically employ two main types of strategies to value and protect their privacy (Chen and Wen, 2020). Findings from this study indicate that middle-aged and elderly users overwhelmingly rely on the first type - preventive privacy protection behavior instead of managing privacy through technical settings. Many participants reported, deliberately reducing or abstaining from posting on WeChat Moments, preferring to share selectively via private messages. Although WeChat offers settings such as “Share only with…” or “Do not share with…,” few participants actively used them. As Park (2013) observed in the research on digital literacy and online privacy behavior, compared with younger users with better new media literacy, the elderly face more significant obstacles in privacy settings than younger people.

Unlike young people who typically use settings such as grouping to control the audience who can access their information, participants in our study reported that they mainly avoid potential risks through more direct and simple methods. This behavior is partly influenced by cultural factors and partly due to the difficulty in managing complex privacy settings. From the perspective of Cognitive Load Theory (Sweller, 2011), complicated privacy settings create a high cognitive burden for older adults, making simple avoidance strategies more appealing than technical adjustments. Participants in our study described that reducing posting or using private messages felt safer. This tendency is further strengthened by cultural values of modesty and self-restraint, which discourage overt self-expression and make “not posting” a socially acceptable form of protection.

the cognitive burden imposed by complicated settings leads them to favor simpler coping strategies when protecting their privacy. This behavior is further reinforced by cultural values of restraint and modesty, which discourage overt self-expression and amplify the preference for avoidance.

Paradoxically, while middle-aged and elderly people exhibit a high level of concern and anxiety about privacy, they are also among the most exposed. Public discourse and media narratives often portray them as vulnerable targets of information leakage and online fraud. This perception is not unfounded: despite their concerns, older users frequently disclose personal information out of necessity. Under digital stress, complete withdrawal from digital life is unrealistic. Many essential services, such as mobile payments, transportation, and healthcare require personal data disclosure. As a result, users experience a tension between fear of exposure and fear of exclusion.

This study reveals that the “privacy paradox” among middle-aged and elderly people is not merely a cognitive inconsistency but a structural dilemma produced by uneven digital literacy and institutional dependence on digital infrastructures. On one hand, they express concern about the leakage of their personal privacy; on the other hand, they have to disclose a large amount of privacy information. In different situations, their privacy concerns and disclosure motivations vary, leading to different privacy attitudes and behaviors (Acquisti et al., 2015).

Beyond psychological motivations such as self-verification, relationship development, and self-presentation (Lee et al., 2013), the paradox is sustained by the practical compulsion to participate in digital systems. As Zhang et al. (2018) demonstrated, while privacy concerns negatively affect information disclosure behaviors among mobile social users, privacy anxiety does not significantly moderate the relationships between perceived ease of use, perceived usefulness, and information disclosure. This may help explain why middle-aged and elderly users are often forced to disclose personal information under digital stress.

Ultimately, the privacy paradox under digital stress reflects a complex interplay between digital stress, cognitive limitations, and cultural values. Rather than simple inconsistency, it represents an adaptive negotiation in which users strive to balance security, convenience, and social inclusion. This research reveal that what appears as attitudinal inconsistency is, in fact, a structural adaptation shaped by digital stress and infrastructural dependence.

### 5.3 Privacy calculus and strategic compromise

For many middle-aged and elderly people, the dilemma is not only rooted in heightened concerns over privacy leakage but is intensified by digital stress. In a society where digitalization has become embedded in everyday life, opting out is rarely feasible. Culnan and Armstrong’s theory of the Privacy Calculus (Culnan and Armstrong, 1999) provides a useful lens to understand the trade-off between privacy protection and digital accessibility. This theory posits that individuals perform a cost-benefit analysis when disclosing information and ultimately decide on the extent and manner of disclosure.

However, unlike Culnan and Armstrong, who measured costs and benefits, participants in our study described a different mechanism, in which digital stress becomes an additional and decisive factor shaping their privacy calculus. In the digital age, many middle-aged and elderly individuals have limited choices. Digital stress has become a critical “weight” that accelerates the adoption of digital tools and technologies, causing “benefits” (i.e., convenience, accessibility) to carry greater significance in the trade-off. Several interviewees noted that when restaurants, transportation, and healthcare require mobile payment or registration, there is “no real choice.” The stress has a significant impact on the privacy calculus of middle-aged and elderly users. They do not disregard privacy protection but are compelled to compromise between privacy risks and digital accessibility. As digitalization permeates every aspect of daily life, from payment, travel to healthcare, digital tools are everywhere, forcing middle-aged and elderly people to make difficult choices between convenience and privacy protection when facing privacy concerns.

Despite holding high levels of concern about privacy at the attitudinal level, most respondents ultimately choose to compromise in their behaviors due to digital stress and limited protective capabilities. However, it is important to note that this compromise is not the result of a lack of privacy awareness. On the contrary, participants generally demonstrated heightened vigilance. but because they lacked effective means to protect themselves, they felt they had “no choice” but to “yield.”

That is to say, although participants showed strong privacy awareness, this awareness rarely translated into effective protection. Many older adults understood privacy threats but lacked the technical capacity to implement protective measures. Within the framework of privacy calculus, this literacy gap distorts risk–benefit evaluations—when users know the risks but cannot mitigate them, they are compelled to accept greater exposure for the sake of convenience or necessity.

This behavioral pattern resonates with the concept of privacy cynicism (Hoffmann et al., 2016), reflecting a state of resigned acceptance where individuals recognize privacy risks but feel powerless to resist them. Among older adults, this cynicism is not rooted in apathy but in digital stress and the perceived futility of technical protection, reinforcing their strategic compromises under constrained agency.

A notable example is the practice of linking bank cards while strictly limiting account balances. This “limit strategy” reflects the risk control awareness of middle-aged and elderly people in the digital payment environment. Such strategic decisions not only reduce the possibility of personal loss but also lower the dependence on account security through “risk diversification.” From an economic perspective, this limit strategy is a typical example of “risk aversion” behavior, that is, protecting personal interests by reducing risk exposure in the face of uncertainty. In the process of digital consumption, middle-aged and elderly people manage and limit their financial exposure on digital platforms by setting limits. This “diversification and limit” strategy not only reflects their perception of digital payment risks but also shows their meticulous operation in privacy calculus: balancing convenience with acceptable levels of risk control.

Therefore, our findings suggest that older adults are better understood as active negotiators seeking a balance between digital stress and privacy concerns. Although their privacy protection behaviors may appear passive or reluctant to outsiders, their compromises are not entirely passive. Instead, it is an adaptation and compromise under the stress of digitalization achieved through a series of strategic choices in “privacy calculus.”

Furthermore, the findings highlight a deeper issue of responsibility attribution in privacy protection. Digital platforms often shift the burden of safeguarding data onto individuals who lack the technical literacy to do so effectively. Pre-ticked consent boxes, complex privacy settings, and opaque data policies create an illusion of autonomy while systematically constraining real choice. Within the logic of privacy calculus, this structural asymmetry distorts users’ cost–benefit evaluations: when meaningful refusal is impossible, disclosure becomes an act of forced consent rather than voluntary exchange.

In our study, results showed that agreeing to data collection is less a choice than a prerequisite for everyday participation. Therefore, privacy protection should be reconceptualized not merely as an individual duty but as a shared responsibility distributed among users, platform designers, regulators, and the broader digital ecosystem. Addressing this imbalance is essential to reduce the structural vulnerability of aging users and to promote more equitable digital inclusion.

## 6 Conclusions and implications

### 6.1 Theoretical implications

This study advances privacy research in three key ways. First, it extends the privacy calculus framework by demonstrating that older adults’ privacy decisions are not driven solely by rational cost - benefit evaluation, but are shaped by digital stress, the pressure to participate in digital society despite limited literacy and high perceived risk. Digital stress functions as a mediating mechanism, explaining why individuals with high privacy concern still choose to disclose information or continue using digital platforms.

Second, the findings enrich the privacy paradox literature by highlighting its cultural impacts. The paradox among Chinese older adults is embedded in collectivist values such as caution, self-restraint, and social harmony. These values influence self-censorship and cautious participation online.

Third, the study highlights the role of intergenerational dynamics. Many participants reported that warnings from adult children increased their fear of digital risks, sometimes reinforcing avoidance or dependence. This suggests that privacy behaviors are relationally constructed, meaning that family communication can mediate or amplify digital stress among older adults.

Taken together, these findings move privacy research beyond individual cognition and toward a more contextualized and culturally informed understanding, integrating emotional, structural, and relational determinants.

### 6.2 Practical implications

In practice, those findings also emphasize that privacy protection should be regarded as a shared responsibility across individuals, technology designers, and policymakers.

For policymakers, privacy literacy interventions could be delivered through culturally familiar and trusted channels. Community-based workshops led by neighborhood committees or senior activity centers, which older adults often regard as authoritative and reliable may be particularly effective. Regulation should also encourage age-friendly authentication and simplified procedures to reduce cognitive load, especially for those who rely on offline interpersonal trust rather than written online instructions.

For technology designers, features such as step-by-step guidance, one-click privacy checks, or human-assisted customer service may reduce anxiety rooted in unfamiliarity and fear of “making mistakes.”

For families, especially adult children, the results show that overprotective warnings may unintentionally amplify fear. Culturally appropriate, autonomy-preserving support, patient teaching, and allowing trial-and-error can help older adults build confidence while maintaining family trust and emotional harmony.

### 6.3 Limitations and future research directions

Despite its contributions, this study has several limitations. First, the qualitative sample was geographically concentrated, which may not fully represent the diversity of older users across China. Second, self-reported data may involve selective memory or social desirability bias. In addition, due to the exploratory nature of the study, certain demographic details such as education and occupation were not systematically collected, which limits the contextual depth of participant profiles. Future research could adopt mixed methods or larger-scale comparative studies to validate these findings. Longitudinal or cross-cultural designs could also explore how intergenerational support and digital literacy training influence privacy behavior over time, offering a broader understanding of aging and digital inclusion.

### 6.4 Conclusion

Although academic attention to privacy issues has increased in recent years, most research focuses on young people or general populations, with relatively few studies addressing older adults. As a group that entered the digital age later, the elderly possess unique perspectives and coping strategies regarding online privacy.

This study explored how middle-aged and elderly users in China perceive and manage privacy under digital stress. Findings reveal that while older adults demonstrate a high level of privacy awareness, their protective behaviors are constrained by cultural norms of restraint, limited digital literacy, and structural dependence on digital infrastructures. These constraints lead to a privacy paradox under digital stress, where individuals remain vigilant yet make strategic compromises to sustain digital participation. Through the lens of privacy calculus, this study shows that such compromises represent rational adaptations rather than passive submission.

## Data Availability

The datasets presented in this article are not readily available because due to ethical and privacy concerns, the dataset is not publicly available but may be shared upon reasonable request and subject to institutional approval. Requests to access the datasets should be directed to JH, haojy1002@gmail.com.

## Appendix A

### Semi-structured focus group interview guide.

– Could you briefly describe how you use smartphones or WeChat in daily life?

– Someone has mentioned concerns about online privacy, what do you think about this? /(If privacy has not been mentioned yet) When people talk about online privacy, what does it mean to you?

– What kinds of privacy risks concern you the most in your daily use of digital tools?

– Do you use mobile payment services? why?

– Have you linked your bank account to online payment platforms? How do you feel about this?

– What do you usually do to protect your personal information online?

– Are there things you choose not to do online because of privacy concerns?

– Have you ever encountered or heard of privacy-related problems ?

– When you worry about privacy or feel unsure, whom do you usually turn to for help?

– Do you feel that using digital tools today is a choice or a necessity? Why?

– Is there anything else you would like to add or share about your experiences with digital life or online privacy?
